# Facile synthesis of 1.3 nm monodispersed Ag nanoclusters in an aqueous solution and their antibacterial activities for *E. coli*†

**DOI:** 10.1039/c8ra04387f

**Published:** 2018-08-28

**Authors:** Chengpeng Jiao, Yuantao Pei, Liqiong Wang, Haijun Zhang, Zili Huang, Yuhuan Dai, Feng Liang, Simin Liu, Yuhua Wang, Shaowei Zhang

**Affiliations:** The State Key Laboratory of Refractories and Metallurgy, Wuhan University of Science and Technology Wuhan 430081 China zhanghaijun@wust.edu.cn +86-27-68862829; Hubei Key Laboratory for Efficient Utilization and Agglomeration of Metallurgical Mineral Resources, Wuhan University of Science and Technology Wuhan 430081 China; Institute of Biology and Medicine, Wuhan University of Science and Technology Wuhan 430065 China; College of Chemical Engineering and Technology, Wuhan University of Science and Technology Wuhan 430081 China; Hubei Province Key Laboratory of Science in Metallurgical Process, Wuhan University of Science and Technology Wuhan 430081 China; College of Engineering, Mathematics and Physical Sciences, University of Exeter Exeter EX4 4QF UK s.zhang@exeter.ac.uk

## Abstract

A facile one-pot strategy was developed to prepare ultrastable monodispersed Ag nanoclusters (NCs) in aqueous solution by using ISOBAM-104, as a stabilizing agent. The as-prepared Ag NCs with an average size of 1.3 nm, which can be preserved in water solution for more than one year under ambient conditions without obvious agglomeration, exhibited excellent antibacterial activities for *E. coli* (DH5α), compared to most of the previously reported results.

## Abstract 2

Ag NCs of 1.3 nm prepared by a facile one-pot strategy exhibit excellent antibacterial activities for *E. coli*.

## Graphical abstract

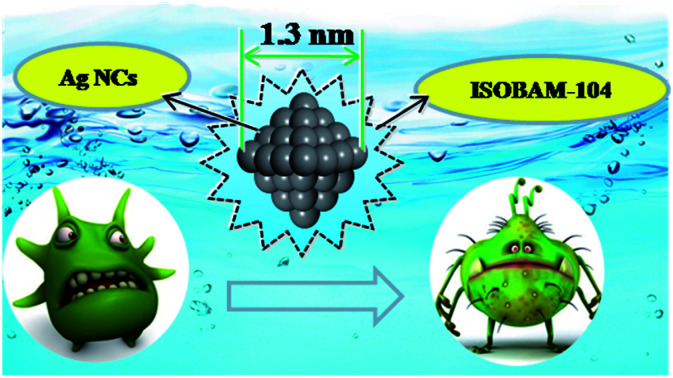

## Introduction

Development of Ag nanoclusters with sizes less than 2 nm has been an attractive research frontier due to their unique fascinating physicochemical properties for application in catalysis, electrochemical devices, plasmonic related technologies or antibacterial agents for killing drug-resistant micro-organisms.^1–6^ Two main strategies, *i.e.*, the reduction method and etching method, have been developed for the preparation of Ag NCs with tiny size. Despite these interesting results, these two methods still suffer from a serious problem: the process is complex and usually requires ligand exchange for the former; moreover, thiols are always used to control the nucleation, growth, and stability of the as-prepared Ag NCs in this case.^7–22^ Nevertheless, it is known that thiols are unfavorable for applications in catalysis and biomedicine.^23–26^ For the latter, special ligands with appropriate etching capacity are usually needed to remove the surface atoms of the pre-formed nanoparticles in the preparation process,^27,28^ and the process is often carried out in biphasic solvent mixtures or organic solvents, for example, at the oil/water interface.^29^ In addition, toxic organic solvents such as carbon tetrachloride or toluene are generally necessary for the techniques.

To date, it is well-known that even polymers such as poly(*N*-vinyl-2-pyrrolidone) (PVP) are effective, commercially available, relatively inexpensive and nontoxic stabilizing agents for the preparation of noble-metal NCs.^30–35^ However, the formation of Ag NCs with an average diameter below 2 nm using polymer as a protecting agent has proven to be a substantial technological challenge.^36,37^ Thus, alternative thiol-free methods using easily accessible polymer as stabilizing agents for the preparation and stabilization of tiny Ag NCs in aqueous environment are of great interest.

Up to now, many metal-containing antibacterial nanoparticles especially Ag nanoparticles have been reported to show pronounced efficiency on deactivating and inhibiting growth of both Gram negative and Gram positive bacteria.^38–40^ At current, a possible mechanism that Ag^+^ with thiol groups in enzymes and proteins is accepted to be the main responsible for the antimicrobial action. And for Ag NPs case, it is also accepted that they can absorb onto cytomembrane of bacteria at first and then entry into cell, result in changes of membrane permeability and damage the intracellular structures of biomolecules, ultimately cause cell death.^41^

Based on above consideration. Here, for the first time, we report a facile one-pot versatile protocol for producing ultrastable monodispersed Ag NCs with an average size of 1.3 nm using poly (isobutylene-*alt*-maleic anhydride) (Fig. S1,† ISOBAM-104, molecular weight: 10^4^ to 10^5^ g mol^−1^) as stabilizing agent in aqueous solution. The as-prepared Ag NCs can be stable stored in ambient condition for at least one year and exhibited excellent antibacterial property for *E. coli* (DH5α). The antibacterial activities of as-prepared Ag NCs were evaluated by microdilution method characterized as minimal inhibitory concentrations (MICs, indicate the ability of inhibiting the growth of bacteria but not necessarily killing them.) and minimal bactericidal concentrations (MBCs, indicating the ability of antibiotics in killing bacteria, defined as the lowest concentration of NPs where the bacterial colony was counted lower than five.) for *Escherichia coli* (*E. coli*).

## Results and discussion

**Fig. 1:**
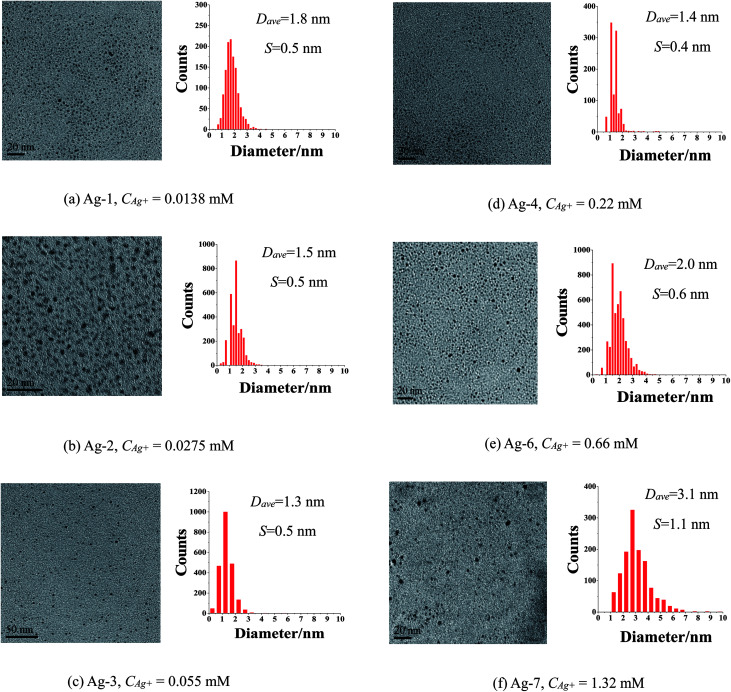
TEM images of as-prepared Ag NCs with various Ag^+^ ion concentrations (AgClO_4_ as precursor, *R*_ISO_ = 40, *R*_KBH_4__ = 2, ice-water bath for 1 h; *D*_ave_: average particle sizes; *S*: standard deviation).

The effect of precursor concentration on size and morphology of Ag NCs was investigated. The UV-Vis spectra (Fig. S2†) of as-prepared Ag NCs consisted of a single surface plasmon resonance (SPR) band at about 400 nm, and the absorbance of the plasmon peak increased with the Ag^+^ ion concentration increasing from 0.0138 mM to 1.32 mM. A series of Ag NCs were prepared by a chemical reduction process with rapid injection of KBH_4_ respectively using AgClO_4_ and ISOBAM-104 as precursor and capping agent (Table S1†). A set of typical TEM images and size distribution histograms shown in Fig. 1 indicated that the mean particle size of Ag NCs was closely dependent on the Ag^+^ ion concentration, and that the average particle diameter initially decreased from 1.8 ± 0.5 nm to the smallest (1.3 ± 0.5 nm) with increasing Ag^+^ ion concentration from 0.0138 to 0.055 mM, and then started to drastically increase to 3.1 ± 1.1 nm upon further increasing the concentration to 1.32 mM. These results suggested that a medium Ag^+^ ion concentration is essential for preparation of Ag NCs with tiny size, this can be explained by nucleation-growth-agglomeration stepwise formation mechanism.^42–44^

**Fig. 2:**
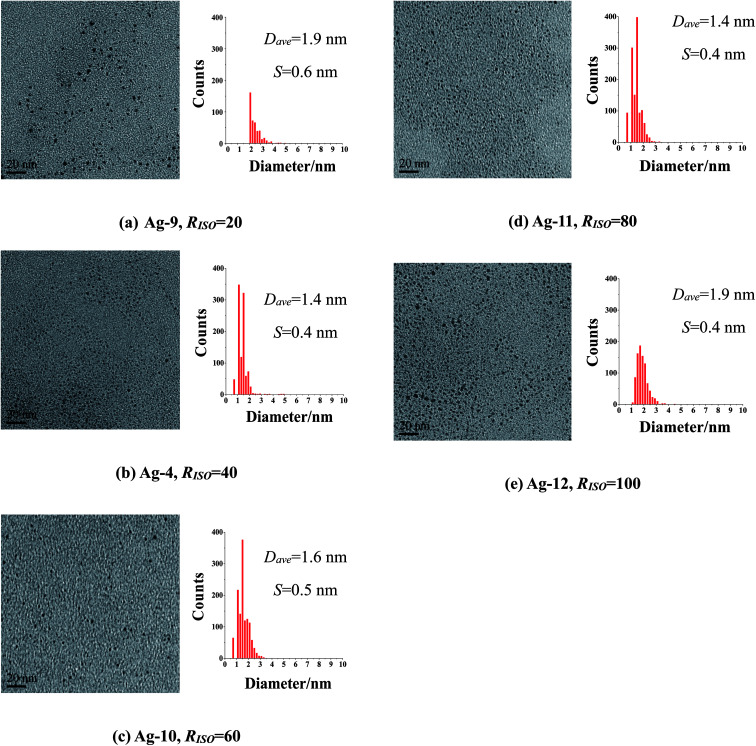
TEM images and size distribution histograms of Ag NCs synthesized at varied *R*_ISO_ ranging from 20 to 100 (AgClO_4_ as precursor, *C*_Ag^+^_ = 0.22 mM, 50 mL, *R*_KBH_4__ = 2, ice-water bath for 1 h; *D*_ave_: average particle sizes; *S*: standard deviation).

Effect of molar ratio of ISOBAM-104 to Ag^+^ ions (denoted as *R*_ISO_) on the preparation of Ag NCs was also investigated. UV-Vis spectra, TEM images and size distribution histograms of as-prepared Ag NCs were shown in Fig. 2. A plasmon characteristic peak around 420 nm of Ag was observed in all the UV-Vis spectra (Fig. S3†), indicating the formation of Ag NCs. Fig. 1 and 2 indicated that all the as-prepared Ag NCs are well-isolated and spherical without any form of aggregation. The size distribution analysis of these prepared NCs indicated that the amount of ISOBAM (*R*_ISO_) did not show clearly effect on the average size of the final Ag NCs, and that the average particle sizes are respectively about 1.9 ± 0.6, 1.4 ± 0.4, 1.6 ± 0.5, 1.4 ± 0.4, and 1.9 ± 0.4 nm for the samples prepared with *R*_ISO_ = 20, *R*_ISO_ = 40, *R*_ISO_ = 60, *R*_ISO_ = 80, and *R*_ISO_ = 100. Moreover, size distribution of Ag NCs (*R*_ISO_ = 40) based on dynamic light scattering (DLS) measurement indicated that the diameter of present NCs was in range of 1–2 nm (Fig. S4†), which was agree with TEM results (Fig. 1). And the zeta potential of present Ag NCs was measured to be −33.7 mV, indicating its excellent stability in this paper. To the best of our knowledge, this is the first report to prepare 1.3 nm Ag nanoclusters in aqueous solution using thiols-free polymer as stabilizing agent.

**Fig. 3:**
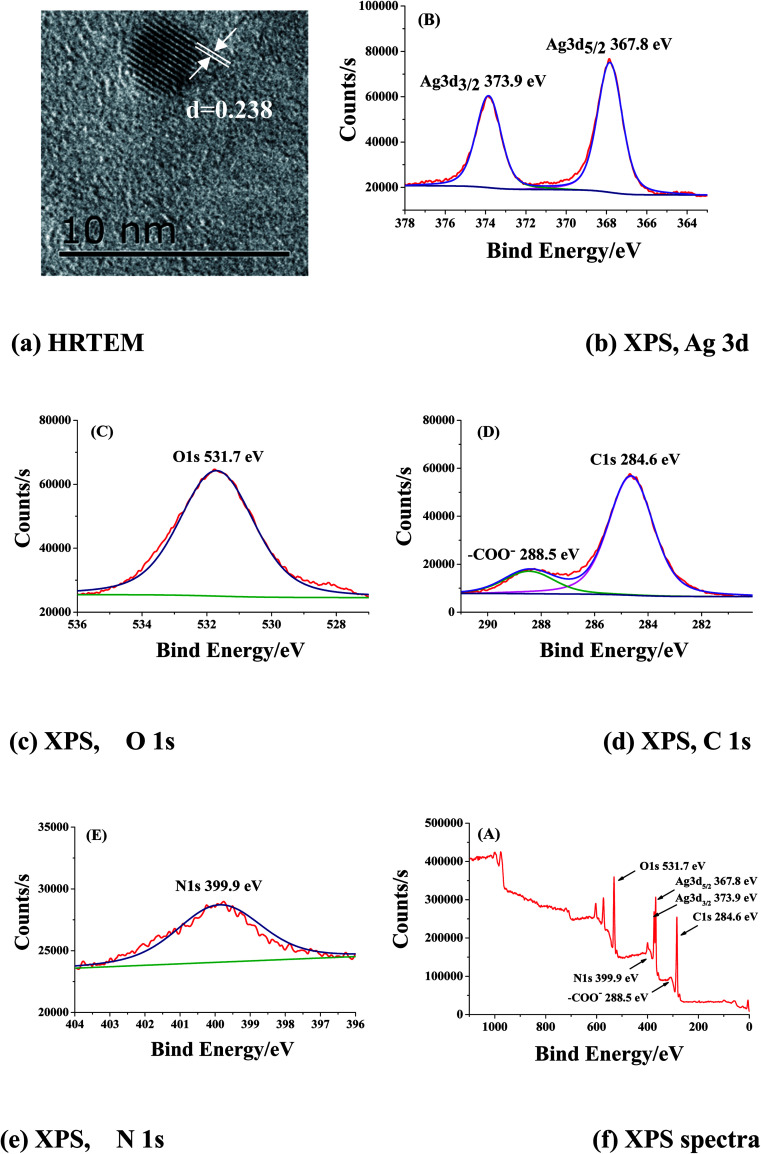
HRTEM images (a) and XPS spectra (b–f) of as-prepared ISOBAM-104 stabilized Ag NCs, Ag 3d (b), O 1s (c), C 1s (d), N 1s (e) and whole range (f) (Ag-4, *C*_Ag^+^_ = 0.22 mM, *R*_ISO_ = 40, *R*_KBH_4__ = 2, ice-water bath for 1 h).

HRTEM was performed to characterize the structure of as-prepared Ag NCs. A typical HRTEM image of a random-chosen Ag NC prepared with *R*_ISO_ = 40 shown in Fig. 3(a) indicated that the interplanar distances of the characterized NC was 0.238 nm, which was well consistent with bulk Ag interplanar spacing of (111) plane (0.2359 nm; Ag: ICCD 00-004-0783).

To furtherly confirm the formation of ISOBAM-stabilized Ag NCs, X-ray photoelectron spectroscopy (XPS) was performed to analyze the element composition of the prepared NCs, a typical XPS spectrum of the Ag NCs was shown in Fig. 3(b–f). Appearance of the binding energy peaks such as Ag 3d, C 1s, O 1s, and N 1s indicates that ISOBAM molecules were indeed adsorbed on the surface of the Ag NCs. Two peaks at 373.9 and 367.8 eV respectively result from Ag 3d_3/2_ and Ag 3d_5/2_ were observed shown in Fig. 3(b), confirming the formation of metal Ag. Moreover, both the electron apparent binding energy (BE) shifted towards a lower binding energy relative to bulk Ag (374.2 eV for Ag 3d_3/2_, 368.2 eV for Ag 3d_5/2_). On the other hand, the BE of O1s in carboxyl was slightly positively shifted from 529.7 eV to 531.7 eV, as shown in Fig. 3(c). These results suggested that there probably present strong electron interactions between the ISOBAM-104 and as-prepared Ag NCs. And the functional group of carboxyl and amide in ISOBAM-104 might act as effective coordination ligands for stability of Ag NCs *via* the electron interaction, which finally made ISOBAM-104 an efficient protective agent for the size control of Ag NCs.

**Fig. 4:**
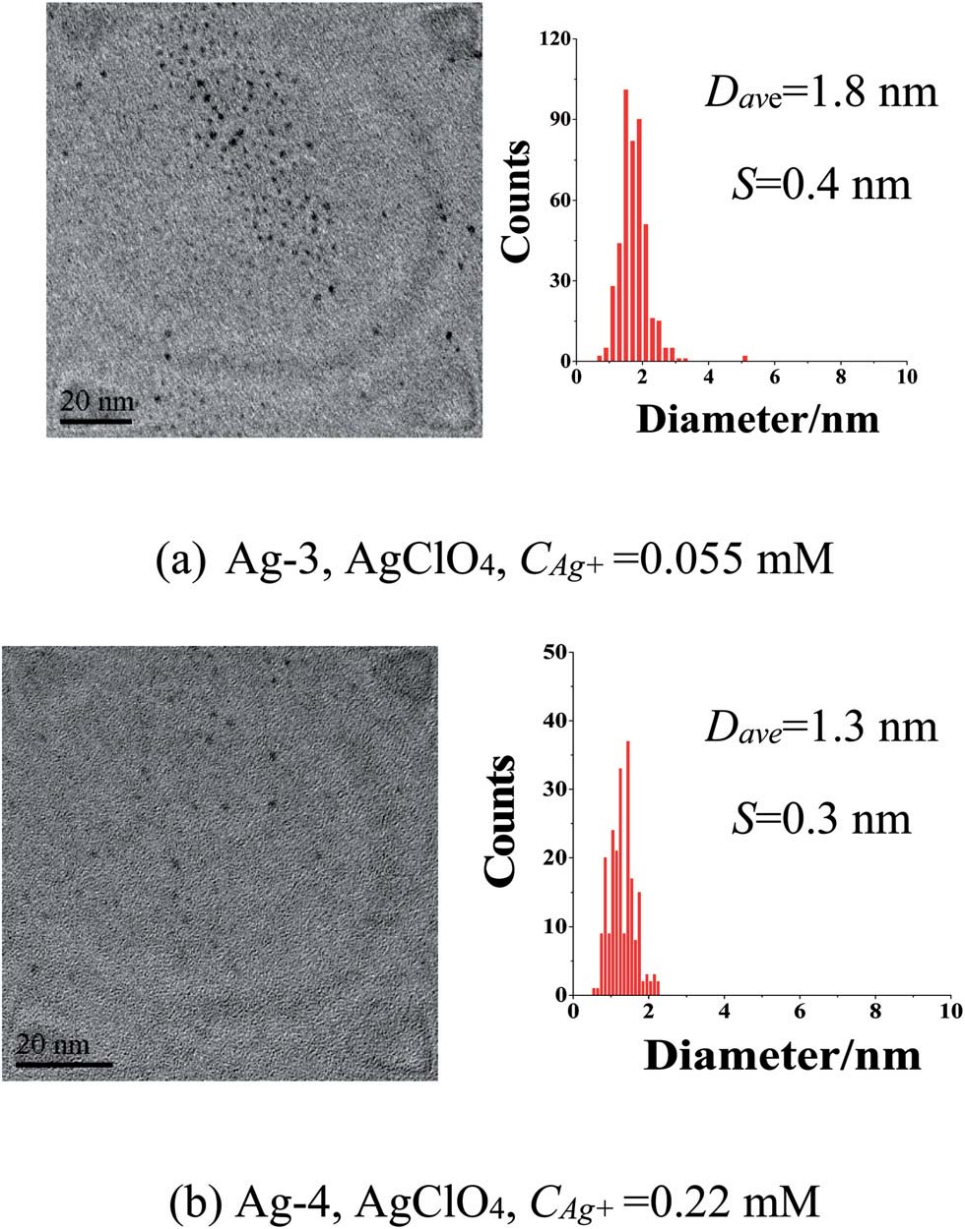
TEM images and size distribution histograms of Ag NCs stored for one year synthesized at varied *C*_Ag^+^_ ranging from 0.055 mM to 0.22 mM. (AgClO_4_, *R*_ISO_ = 40, *R*_KBH_4__ = 2, reduced under ice bath for 1 h; *D*_ave_: average particle sizes; *S*: standard deviation)

For comparison, PVP (K30, average molecular-weight of about 30 000), a widely used polymer for metal nanocluster preparation was also chosen as stabilizing agent to synthesize Ag nanoparticles using rapid injection and dropwise addition of KBH_4_ method, alcohol reduction method and sodium citrate reduction method, the results showed that most of the prepared Ag nanoparticles exhibited much larger average particle size and wider size distribution, and some of them showed irregular morphology (Fig. S5†). Moreover, a comparison of Ag nanoparticles prepared with different protecting agents listed in Table S2† also indicated that the Ag NCs prepared in present paper has the smallest average size. These results demonstrated that ISOBAM is indeed an efficient stabilizing agent for preparation of Ag NCs with tiny size. What should be noted is that the average particle size of prepared Ag NPs synthesized with various *C*_Ag^+^_ ranging from 0.055 mM to 0.22 mM did not show obvious growth even after storage under ambient conditions for one year (Fig. 4). For the former sample prepared with *C*_Ag^+^_ = 0.055 mM whose average diameter changed from 1.3 ± 0.5 nm to 1.8 ± 0.4 nm after one year; and the latter one changing from 1.4 ± 0.4 nm to 1.3 ± 0.3 nm. Furthermore, it showed that the new stabilizing agent also makes it possible to prepare other metal NCs with tiny size, such as Au, Pt, Rh, and so on (Fig. S6†).

One important application of Ag NCs is the potential application as antibiotics, thus, antibacterial activities characterized as minimal inhibitory concentrations (MICs) and minimal bactericidal concentrations (MBCs) for *Escherichia coli* (*E. coli*) of as-prepared Ag NCs were evaluated by microdilution method according to the reported method.^45,46^ MIC was initially confirmed by average OD_600_ absorbance results of *E. coli* in 96-well microplate after incubating on a shaker bed at 200 rpm under 37 °C overnight, and MBC was confirmed by reinoculating 10 μL of each bacterial suspension after MIC test on nutrient agar plates at 37 °C for 10 h, 24 h and even 36 h (Fig. S7†). All the tests for MIC and MBC were performed at least three times. It showed that the as prepared Ag NCs did not show obvious disturbance on OD_600_ test under the usage concentration below 1.35 mg L^−1^ (more OD_600_ results were shown in Table S3†). Moreover, the protective agent ISOBAM-104 did not show any inhibitory effects, and high ISOBAM-104 concentration, for example, 0.75 mg L^−1^, even promoted the growth of *E. coli*, which can be concluded *via* the higher OD_600_ results of 0.770 than that of control group at 0.739 after cultivating for 24 h under 37 °C (shown in Table S4†). It also turns out that Ag NCs (Ag-4, *C*_AgClO_4__ = 0.22 mM, 50 precursor solution, *R*_ISO_ = 40) with an average particle size of about 1.4 ± 0.4 nm showed excellent antibacterial activities with MIC and MBC as low as 0.25 mg L^−1^ and 0.50 mg L^−1^ (shown in Tables 1 and S4†). The stability of present Ag NPs (Ag-4) in NaCl solutions was also investigated, and 4 mL as prepared Ag-4 NC colloid was mixed with 4 mL NaCl solution with different concentrations, the photos of the mixture after 5 min to 24 h were shown in Fig. S8.† Although it indicated that the color fading became severe with the NaCl concentration increase, however, the sample no. 5^#^ which share an identical NaCl concentration for antibacterial experiment show negligible color fading compared with the blank control, and can be stably stored for 24 h. Since the mole ratio of NaCl : Ag in the sample is as high as 1545 : 1, it can be concluded that present Ag NCs are very stable in NaCl solution. TEM was used to characterize the morphology of present Ag NCs in NaCl solution, and it confirmed again that the Ag NCs were well dispersive. (Fig. S9†).

**Table 1:** Antibacterial activity for *E. coli* of as-prepared ISOBAM-protected Ag NCs^a^

Code	Preparation conditions and size of NCs			Antibacterial activities	
	*C*_Ag^+^_, mM	*R*_ISO_	Average size, nm	MIC, mg L^−1^	MBC, mg L^−1^
Ag-2	0.0275	40	1.5 ± 0.5	0.50	0.75
Ag-3	0.055	40	1.3 ± 0.5	0.50	0.75
Ag-4	0.22	40	1.4 ± 0.4	0.25	0.50

a *C*_Ag^+^_ refers to the concentration in 50 mL precursor solution for NCs preparation.

To the best knowledge of the authors, these antibiotic activities are the lowest reported so far for Ag NCs, and the excellent antibiotic activity for *E. coli* of as-prepared Ag NCs is believed to be ascribed to its tiny size because it is demonstrated that the interaction of silver NCs with light or microorganisms can be manipulated by its size and capping agents.^47,48^ Moreover, a comparison between the antibiotic activity of the present Ag NCs for *E. coli* and that of NCs reported in literature is given in Table S5.†

## Conclusion

In summary, a facile one-pot versatile approach for large-scale fabrication of monodispersed and spherical Ag NCs with size of 1.3 nm in aqueous solution was developed *via* using a novel thiols-free copolymer of ISOBAM-104 as stabilizing agent. The as-prepared Ag NCs stabilized by the polymer showed well stability and can be preserved in water for more than one year under ambient condition without changing its particle size. These NCs exhibits excellent antibacterial activity and lowest MIC and MBC so far reported for *E. coli* (DH5α). We believe that our finding in present paper not only extend the preparation method of Ag NCs with size less than 2.0 nm, but also provide a new protective agent for synthesis of metal NCs.

## Funding sources

This work was financially supported by the National Natural Science Foundation of China (Grant No. 51472184 and 51472185), and Program for Innovative Teams of Outstanding Young and Middle–aged Researchers in the Higher Education Institutions of Hubei Province (T201602), and the Key Program of Natural Science Foundation of Hubei Province, China (2017CFA004).

## Conflicts of interest

The authors declare that they have no conflict of interest.

## Supplementary Material

RA-008-C8RA04387F-s001
