# Supplementary material for: Facile synthesis of 1.3 nm monodispersed Ag nanoclusters in an aqueous solution and their antibacterial activities for E. coli
Source: RSC Adv. 2018 Aug 28;8(53):30207–14. doi: 10.1039/c8ra04387f (PMC9085391; doi:10.1039/c8ra04387f)
Supplement: RA-008-C8RA04387F-s001 [file RA-008-C8RA04387F-s001.pdf]

Electronic Supplementary Material (ESI) for RSC Advances.
This journal is © The Royal Society of Chemistry 2018

Supporting Information

Facile synthesis of 1.3-nm monodispersed Ag nanoclusters in an aqueous solution and its antibacterial activities for E. coli

Chengpeng Jiao[1,2,‡], Yuantao Pei[1,‡], Liqiong Wang[1], Haijun Zhang[1], Zili Huang[2], Yuhuan Dai[3], Feng Liang[4], Simin Liu[4], Yuhua Wang[5], Shaowei Zhang*[6]*

1. The State Key Laboratory of Refractories and Metallurgy, Wuhan University of Science and Technology, Wuhan 430081, China.

2. Hubei Key Laboratory for Efficient Utilization and Agglomeration of Metallurgical Mineral Resources, Wuhan University of Science and Technology, Wuhan 430081, China.

3. Institute of Biology and Medicine, Wuhan University of Science and Technology, Wuhan 430065, China.

4. College of Chemical Engineering and Technology, Wuhan University of Science and Technology, Wuhan 430081, China.

5. Hubei province Key Laboratory of Science in Metallurgical Process, Wuhan University of Science and Technology, Wuhan 430081, China

6. College of Engineering, Mathematics and Physical Sciences, University of Exeter, Exeter EX4 4QF, UK.

‡ Chengpeng Jiao and Yuantao Pei contributed equally to this work.

*Corresponding author, Prof. Dr. Haijun Zhang, Fax: +86-27-68862829, E-mail: zhanghaijun@wust.edu.cn. Prof. Dr. Shaowei Zhang, E-mail: s.zhang@exeter.ac.uk

Experimental Section

Chemicals: Silver perchlorate ($AgClO_4$, 97%), silver nitrate ($AgNO_3$, 99.8%) were purchased form Aldrich. Potassium borohydride (KBH_4, 95.0%), PVP (K30, average molecular weight of about 30, 000), **trisodium citrate** ($C_6H_5Na_3O_7{\cdot}2H_2O$) and ethanol ($C_2H_5OH$, 99.7%) were purchased from Sinopharm Ltd.. ISOBAM purchased from Aldrich is the trade name of an alternative copolymer of isobutylene and maleic anhydride developed by KURARAY using their POVAL (polyvinyl alcohol) technology, and ISOBAM-104 (CAS NO. 52032-17-4) is amide-ammonium salt types of ISOBAM. All chemicals were directly used as raw materials without further purification.

Synthesis of Ag nanoclusters (NCs) using ISOBAM-104 or PVP as capping agent by a rapid injection of KBH_4

In a typical synthesis process, 50 mL aqueous solution of ISOBAM-104 (8.8 mM, the molar ratio of ISOBAM-104 monomer unit to $AgClO_4$ is 40, denoted as R_{ISO} = 40) was mixed with $AgClO_4$ (50 mL, 0.22 mM) solution in a 250 ml two neck round-bottom flask. Then the mixture was placed in the ice-water bath with flowing N_2 for 30 min under vigorous magnetic stirring. After that, an aqueous solution of KBH_4 (10 mL, 2.2 mM, 0 °C; R_{KBH4} is defined as the molar ratio of $NaBH_4$ to the total Ag^+ ions.) was rapidly injected into the mixture under vigorous stirring within 3 s, followed by a mixing for another 1 hour. The detailed processing conditions are listed in Table S1.

Synthesis of Ag nanoparticles using PVP as capping agent by dropwise addition of KBH_4

Typical, $NaBH_4$ (20 mL) was dropwise added into the Ag^+/PVP aqueous solution in an ice-water bath at 0 °C, the addition time of $NaBH_4$ was 1 hour; after that, the mixture was mixed for another 4 h.

Synthesis of Ag nanoparticles using PVP as capping agent by alcohol reduction method

Typical, an aqueous solution of $AgClO_4$ (50 mL, 1.32 mM, ethanol / water = 1/4 (v/v)) was added into a PVP (50 mL, 132 mM in monomer unit, ethanol / water = 1/4 (v/v)) solution and then stirred at room temperature for 15 min. The mixed solutions were stirred and heated to reflux at 100 °C for another 2 h.

Synthesis of Ag nanoparticles using PVP as capping agent by citrate reduction method

Typical, an aqueous PVP solution (the molar ratio of PVP monomer unit to $AgClO_4$ is 40, denoted as R_{PVP} = 40) was mixed with another $AgClO_4$ solution at firstly, and then sodium citrate (the molar ratio of sodium citrate to Ag^+ ions is 10, denoted as $R_{Na3Cit.}$) was added into the mixture in a two neck round-bottom flask in oil-bath at 100 °C and mixed for another 1 h in a N_2 atmosphere.

Characterization: The UV-visible absorbance spectra over 200-800 nm and OD_{600} test were recorded on UV-3600 equipped with a quartz cell with an optical path length of 10 mm and Bio UV-visible spectrophotometer with 96-well microplate, respectively. Dynamic light scattering (DLS) and zeta potential were detected using Nicomp 380 Z3000 at 23 °C and E-Field strength of 4 V/cm. Transmission electron microscopy (TEM) images were taken at the accelerated voltage of 80 k*V* using a FEI Tecnai G2 50-S-TWIN TEM. The samples were obtained by dropping several droplets of the prepared colloidal aqueous solution onto microgrid covered with a thin amorphous carbon film and followed by evaporating at room temperature, generally at least 400 particles from different parts on the grid were selected to calculate average particle size. High-resolution transmission electron microscopy (HR-TEM) images were taken at the accelerated voltage of 200 k*V* using a JEM-2100F Field Emission High-resolution TEM. X-ray photoelectron spectroscopy (XPS) measurement was performed using a Quantum 2000 spectrometer with Al Kα radiation. Binding energies (BE) were charge normalized by taking the C (1s) BE of adventitious carbon

contamination as 284.6 eV, and the analyses on Ag were based on $Ag3d_{5/2}$ and $Ag3d_{3/2}$ peaks.

Antibacterial activity test. The experiments were conducted according to the reported minimal inhibition concentrations (MIC) and minimal bactericidal concentrations (MBC) methods [1,2]. Twofold serial dilutions of Ag NCs colloid with varying Ag concentrations from 0 to 1.00 mg/L were prepared in tubes by using sterile distilled water and then transferred into 96-well microplate. Then freshly prepared microbial suspensions were pipetted into each well in an equal volume. All tests were performed in duplicate.

For MIC, bacteria were cultured in the Liquid broth (LB) medium at 37 ℃ on a shaker bed at 200 rpm until the logarithmic phase (the original bacterial concentration is 10^7 colony-forming units (CFU/mL), inoculation bacterial suspension was obtained by 1:100 dilution). Then 50 μL of Ag NCs with different mass concentration were added into 50 μL condensed LB medium in a 96-well microplate to form 100 μL LB medium. And followed by addition of 10 μL bacterial suspension (the final density of bacteria in each well was 10^4 CFU/mL), and further incubated at 37 ℃ overnight. The MIC was the NC concentration that the optical density of bacterial suspension at 600 nm (OD_{600}) showed no obvious increase, and no visible bacteria grew overnight at the concentration. To rule out the effect of Ag NCs on OD_{600}, the Ag NC concentration for antibacterial test should be kept less than 1.35 mg/L since high usage of Ag NCs will result in obvious absorbance and then increase the OD_{600} values (Table S3).

MBC was confirmed by reinoculating 10 μL of each bacterial suspension after MIC test on nutrient agar plates at 37 °C for 10 h, 24 h and even 36 h (see images in Fig. S6). MBC was the lowest concentration of NCs where the bacterial colony was counted lower than five. An equal volume of water pretreated by autoclaving under 120 ℃ for 30 min was added to that of the aqueous solution of NCs for total volume control or to the LB medium for control. All the tests for

MIC and MBC were performed at least three times.

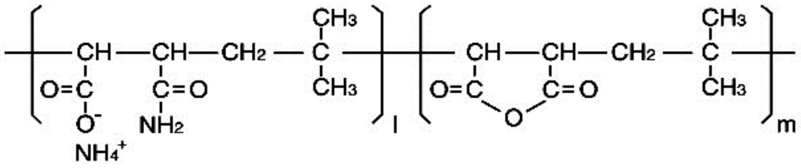

Figure S1 Chemical structure of ISOBAM-104.

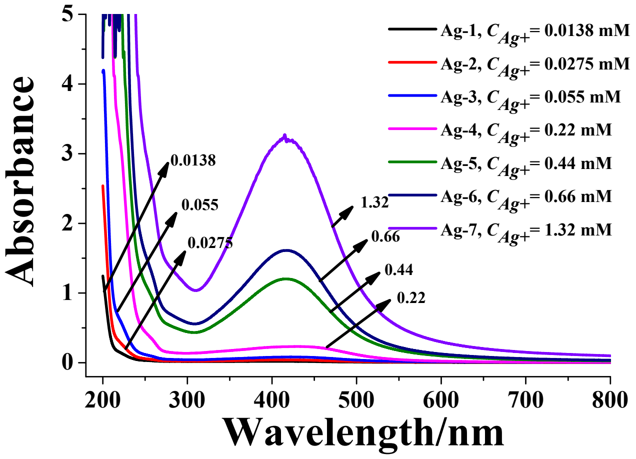

Figure S2 UV-Vis spectra of Ag NCs synthesized at varied C_{Ag+} ranging from 0.0138 to 1.32 mM ($AgClO_4$ as precursor, R_{ISO} = 40, R_{KBH4} = 2, in ice-water bath for 1h.).

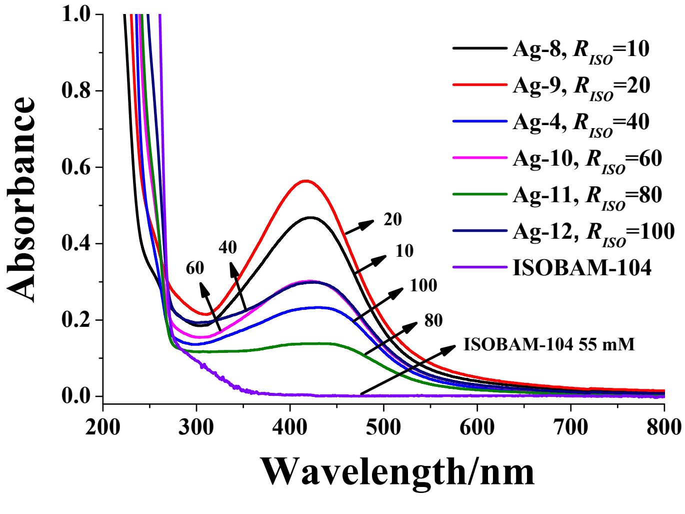

Figure S3 UV-Vis spectra of colloidal Ag NCs synthesized at varied R_{ISO} ranging from 10 to 100 ($AgClO_4$,

CAg+ = 0.22 mM, RKBH4 = 2, icy bath for 1 h).

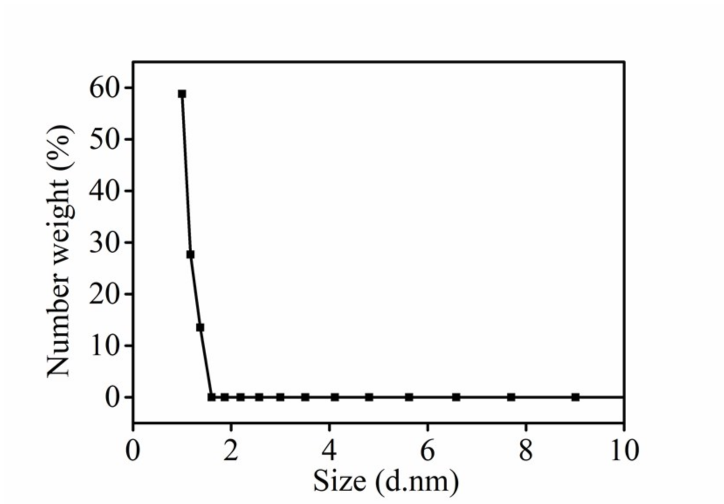

Figure S4 Dynamic light scattering size distribution of Ag NCs prepared with 0.22 mM Ag ions and R_{ISO} = 40 measured at room temperature.

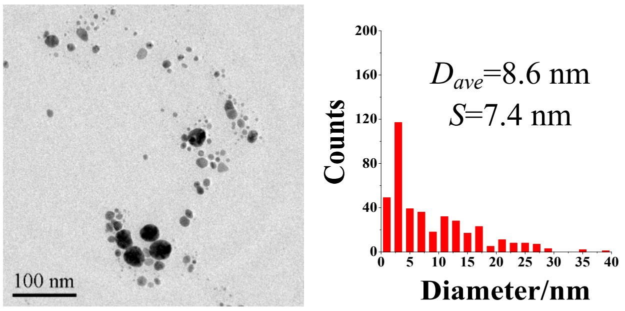

(a) Rapid injection of KBH_4

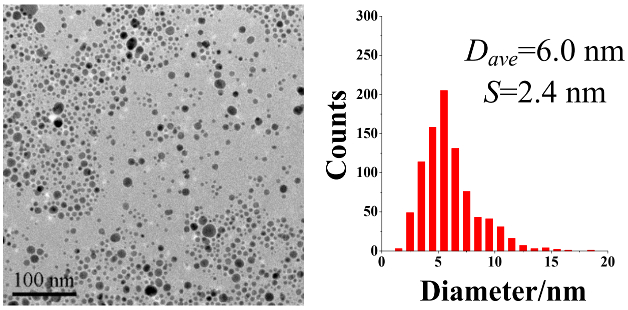

(b) Dropwise addition of KBH_4

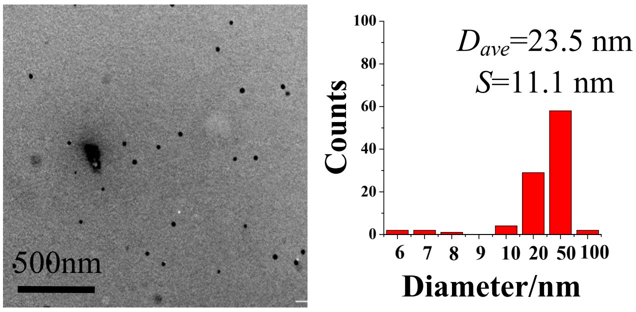

(c) Alcohol reduction

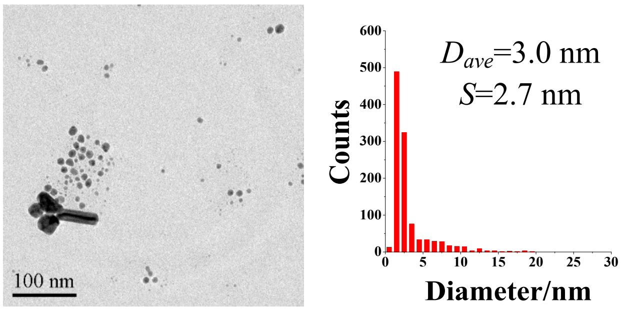

(d) Sodium citrate reduction

Figure S5 TEM images and size distribution histograms of Ag nanoparticles synthesized using PVP as capping agents: (a) rapid injection of KBH_4 ($AgClO_4$ as precursor, R_{PVP} = 100, C_{Ag+} = 0.44 mM, R_{KBH4} = 5, reduced under ice bath for 1 h), (b) dropwise addition of KBH_4 ($AgClO_4$ as precursor, R_{PVP} = 20, C_{Ag+} = 1.32 mM, R_{KBH4} = 5, reduced under ice bath for 1 h), (c) alcohol reduction ($AgClO_4$ as precursor, R_{PVP} = 100, C_{Ag+} = 0.66 mM, reduced in oil bath at 100 ℃ for 2 h, C_2H_5OH/H_2O=1/9 (V/V)), (d) sodium citrate reduction ($AgClO_4$ as precursor, R_{PVP} = 40, C_{Ag+} = 1.98 mM, $R_{Na3Cit.}$ = 10, reduced in oil bath at 100 ℃ for 1 h); (D_{ave}: average particle sizes; S: standard deviation.).

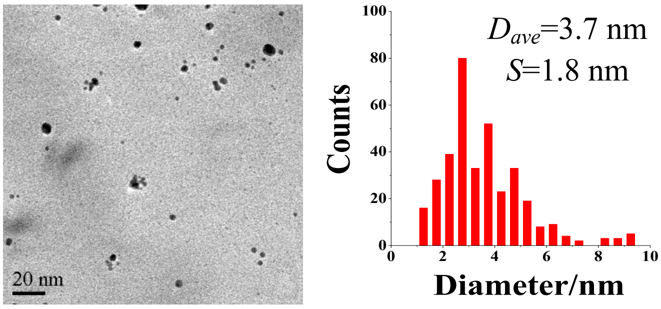

(a) Au

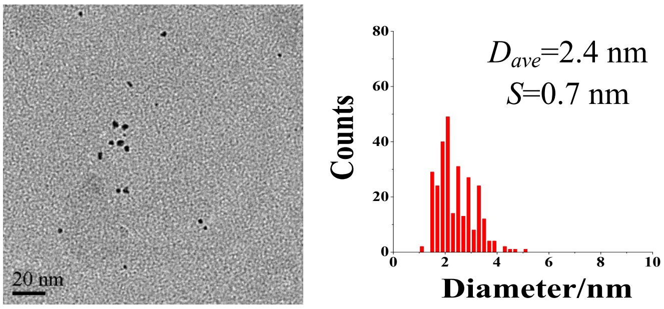

(b) Pt

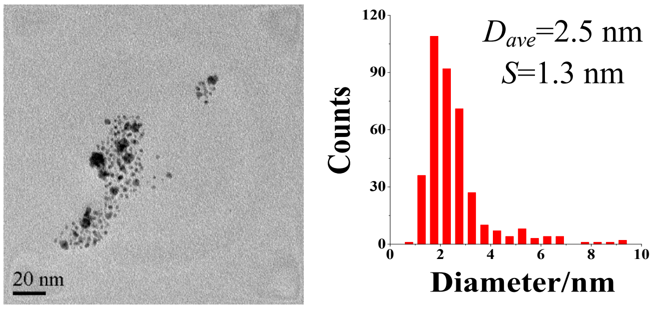

(c) Rh

Figure S6 TEM images and size distribution histograms of Au, Pt, Rh NPs synthesized using ISOBAM-104 as protective agents. (R_{ISO} = 40, C_{metal} = 0.22 mM, R_{KBH4} = 5, reduced under ice bath for 1 h; D_{ave}: average particle sizes; S: standard deviation.).

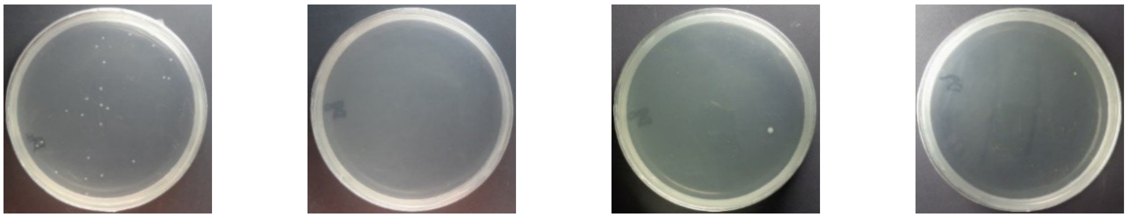

0.50 mg/L, 10 h   0.75 mg/L, 10 h   0.75 mg/L, 24 h   0.75 mg/L, 36 h

(a) Ag-7, C_{Ag+} =1.32 mM

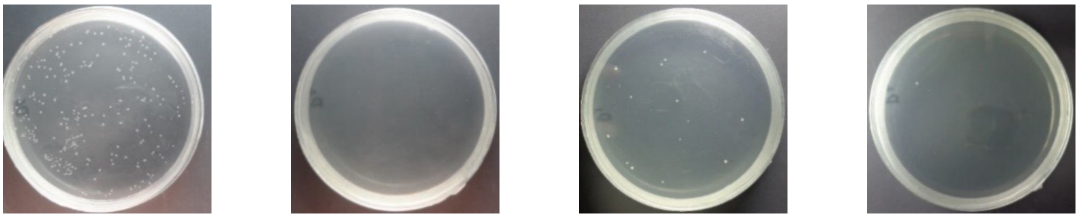

0.50 mg/L, 10 h   0.75 mg/L, 10 h   0.75 mg/L, 24 h   0.75 mg/L, 36 h

(b) Ag-6, C_{Ag+} =0.66 mM

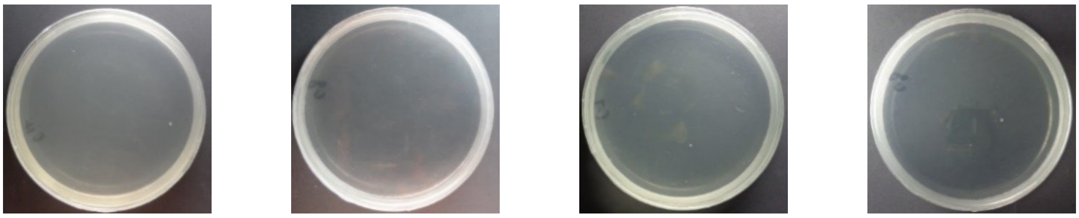

0.25 mg/L, 10 h   0.50 mg/L, 10 h   0. 50 mg/L, 24 h   0. 50 mg/L, 36 h

(c) Ag-4, C_{Ag+} =0.22 mM

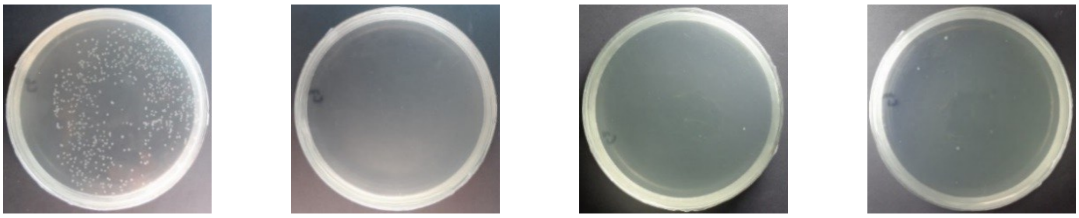

0.50 mg/L, 10 h   0.75 mg/L, 10 h   0.75 mg/L, 24 h   0.75 mg/L, 36 h

(d) Ag-3, C_{Ag+} =0.055

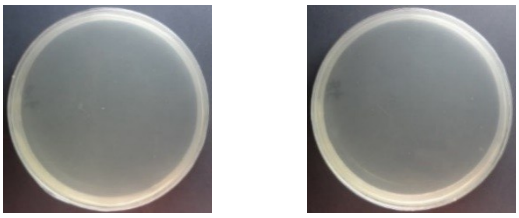

0.50 mg/L, 10 h   0.75 mg/L, 10 h

(e) Ag-2, C_{Ag+} =0.0275 mM

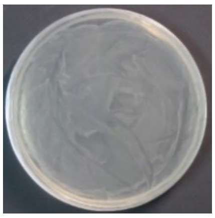

(f) LB broth, 36 h

Figure S7 Dependence of precursor concentration of Ag^+ ions (with unit of mM), dosage of Ag NCs (with unit of mg/L) for antibacterial test and time on MBC test (on LB agar plates at 37 ℃, C_{Ag+} refers to the concentration for Ag NC preparation) : (a) Ag-7, C_{Ag+} = 1.32 mM, (b) Ag-6, C_{Ag+} = 0.66 mM, (c) Ag-4, C_{Ag+} = 0.22 mM, (d) Ag-3, C_{Ag+} = 0.055 mM, (e) Ag-2, C_{Ag+} = 0.0275 mM, (f) LB broth without Ag NCs.

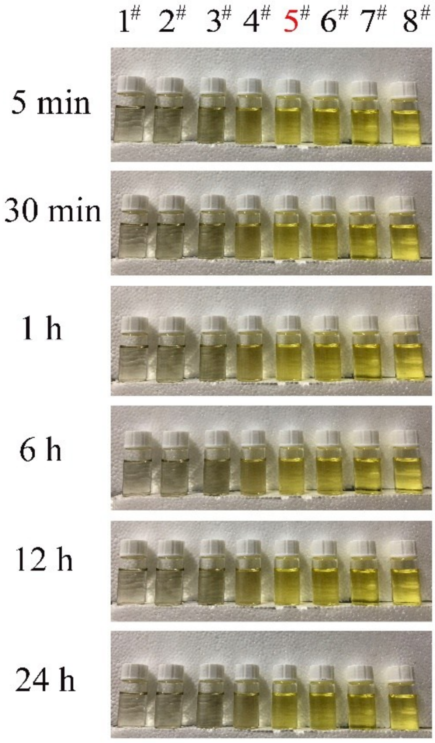

Figure S8 Photos of Ag-4 NC colloid (4 mL) mixed with NaCl solution (4 mL) with different concentration after 5 min~24 h. No.1#~7# containing 2.74, 1.37, 0.68, 0.34, 0.17, 0.09, 0.04, 0.02 mol/L NaCl aqueous solution and No.8 is blank control.

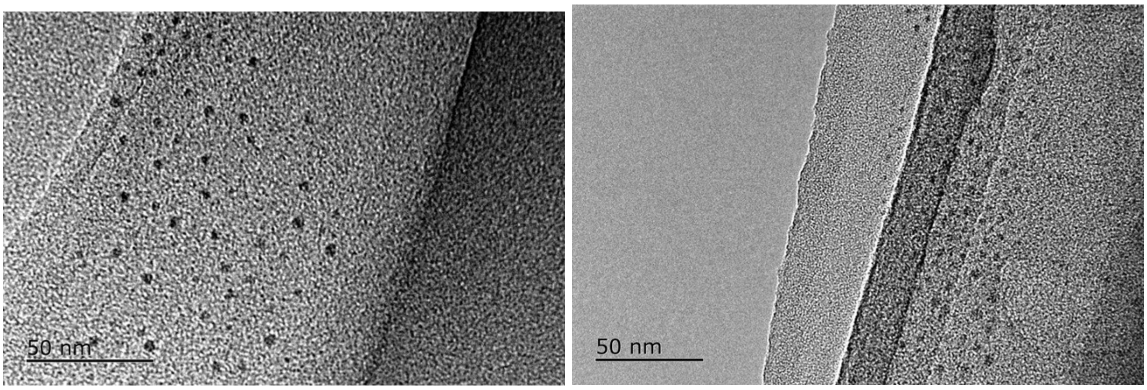

Figure S9 TEM images of Ag-4 sample mixed with 0.17 mol/L NaCl solution after 24 h.

Table S1 Batch compositions and processing conditions for Ag NC preparation

Codes	Concentration of Ag ions/(mM)	R_{ISO}	Reaction time /h	Reaction temperature/°C
Ag-1	0.0138			
Ag-2	0.0275			
Ag-3	0.055			
Ag-4	0.22	40		
Ag-5	0.44			
Ag-6	0.66			
Ag-7	1.32		1	0
Ag-8		10		
Ag-9		20		
Ag-10	0.22	60		
Ag-11		80		
Ag-12		100		

Table S2 Comparison of average particle size of Ag nanoparticles synthesized using different protective agent

	Protective agent	Average size /nm	Solvent	Method	Reference
Ag nanoparticles	Tannic acid	10.0	water	Citrate and tannic acid reduction	[3]
Ag nanoparticles	α-, β-, γ-cyclodextrins	0.5~7.0	water	Citrate acid reduction	[4]
Ag nanoparticles	Dextran	5.0~10.0	water	Dextran reduction	[5]
Ag nanoparticles	CTAB	10.0~30.0	water	Aniline reduction	[6]
Ag nanoparticles	Cochlospermum gossypium	5.5 ± 2.5	water	Cochlospermum gossypium reduction	[7]
Ag nanoparticles	Daxad 19	4.5 ± 2.9	water	Polyethylene glycol and Daxad 19 reduction	[8]
Ag nanoparticles	PVP	7	water	$NaBH_4$ reduction	[9]
Ag nanoparticles	PAN	3	water	$NaBH_4$ reduction	[9]
Ag nanoparticles	L -cys	10	water	$NaBH_4$ reduction	[9]
Ag nanoparticles	Oleic acid	5	water	$NaBH_4$ reduction	[9]
Ag nanoclusters	ISOBAM-104	1.3±0.5	water	KBH_4 reduction	Present paper

Table S3 OD_{600} results of Ag NC under varied usage concentration

Sample	Preparation conditions of Ag NCs		Conditions for antibacterial activity	OD_{600} under various dosage of Ag NCs		
	C_{Ag+}	R_{ISO}		5.40 mg/L	2.70 mg/L	1.35 mg/L
Ag-6	0.66 mM	40	With *E. coli*	0.202	0.138	**0.077**
Ag-7	1.32 mM			0.213	0.119	**0.079**
ISOBAM-104	55 mM	-		0.436	0.446	0.460
Ag-6	0.66 mM	40	Without *E. coli*	0.178	0.128	**0.072**
Ag-7	1.32 mM			0.200	0.128	**0.064**
ISOBAM-104	55 mM	-		0.045	0.040	0.040
Control	-	-	LB+*E. coli*	0.229		

(OD_{600}: Optical density of bacterial suspension at 600 nm; the OD_{600} results of bacterial suspension for samples Ag-6 and Ag-7 with NC dosage of 1.35 mg/L (in red color) were lower than that with NC dosage of 2.70 mg/L and 5.40 mg/L, which suggested that Ag NC dosage for antibacterial test should be less than1.35 mg/L because high amount of Ag NCs could cause clearly absorbance increase at 600 nm of the suspension, and then result in large experiment error.)

Table S4 OD_{600} results for MIC of Ag NCs used at varied concentration

Sample	Preparation conditions of Ag NCs		Size of prepared Ag NCs/nm	OD_{600} under various dosage of Ag NCs		
	C_{Ag+}	R_{ISO}		0.75 mg/L	0.50 mg/L	0.25 mg/L
Ag-2	0.0275 mM		1.5±0.5	0.063±0.007	0.052±0.004	0.307±0.016
Ag-3	0.055 mM		1.3±0.5	0.052±0.001	0.056±0.001	0.471±0.009
Ag-4	0.22 mM	40	1.4±0.4		0.057±0.001	0.055±0.004
Ag-6	0.66 mM		2.0±0.6	0.055±0.002	0.057±0.001	0.307±0.016
Ag-7	1.32 mM		3.1±1.1	0.079±0.003	0.056±0.003	0.471±0.009
ISOBAM-104	55 mM		--	0.770±0.016	0.733±0.004	
Control	--		LB+*E. coli*		0.739±0.041	

Table S5 Comparison of MIC and MBC of Ag nanoparticles with various particles size

Sample	MIC mg/L	MBC mg/L	Capping agents	Average physical size /nm	Methods of synthesis	Bacteria
Graphene oxide supported Ag NPs	2.5 to 5.0	-	Sodium citrate	7.5	Sodium citrate reduction, 130◦ C /30 min [10]	E. coli
Silica supported Ag NPs	1.0	-	-	4.0	Flame spray pyrolysis [11]	E. coli
Ag NPs	75	-	Oleylamine	8.07±0.02	Oleylamine reduction [12] 200◦ C /30 min, then 150◦ C /4 h	B. megaterium
Ag NPs	2.0	-	Oleic acid	9 to 10	$[Ag(NH_3)_2]^+_{(aq)}$ by glucose reduction with UV irradiation [13]	E. coli and S. aureus
Polyethylenimine -functionalized Ag NPs	2.48	4.96	Polyethylenimine	23.5±3	Hydrothermal process, PEI as reducing and stabilizing agent[14]	E. coli
**Ag NCs in present paper**	**0.25**	**0.50**	**ISOBAM-104**	**1.4±0.4**	**KBH_4 reduction**	**E. coli**

References

1. Zhao, Y. Y., Tian, Y., Cui, Y., Liu, W. W., Ma, W. S. & Jiang, X. Y. Small molecule-capped gold nanoparticles as potent antibacterial agents that target Gram-negative bacteria. *J. Am. Chem. Soc.* **132**, 12349-12356 (2010).

2. Zhao, Y. Y., Ye, C. J., Liu, W. W., Chen, R. & Jiang, X. Y. Tuning the composition of AuPt bimetallic nanoparticles for antibacterial application. *Angew. Chem. Int. Ed.* **53**, 8127-8131 (2014).

3. Cheng, Y., Wang, F., Fang, C., Su, J. & Yang, L. Preparation and characterization of size and morphology controllable silver nanoparticles by citrate and tannic acid combined reduction at a low temperature. *J. Alloy. Compd.* **658**, 684-688. (2016).

4. Suárez-Cerda, J., Nuñez, G. A., Espinoza-Gómez, H., & Flores-López, L. Z. A comparative study of the effect of α-, β-, and γ-cyclodextrins as stabilizing agents in the synthesis of silver nanoparticles using a green chemistry method. *Sci. Eng. C-Mater.* **43**, 21-26 (2014).

5. Bankura, K. P., Maity, D., Mollick, M. M., Mondal, D., Bhowmick, B., Bain, M. K. & Chattopadhyay, D. Synthesis, characterization and antimicrobial activity of dextran stabilized silver nanoparticles in aqueous medium. *Carbohyd. Polym.* **89**, 1159-1165. (2012).

6. Khan, Z., Al-Thabaiti, S. A., Obaid, A. Y., & Al-Youbi, A. O. Preparation and characterization of silver nanoparticles by chemical reduction method. *Colloid. Surface. B. Biointerfaces*, **82**, 513-517. (2011).

7. Vinod, V. T. P., Saravanan, P., Sreedhar, B., Devi, D. K., & Sashidhar, R. B. A facile synthesis and characterization of Ag, Au and Pt nanoparticles using a natural hydrocolloid gum kondagogu (Cochlospermum gossypium). *Colloid. Surface. B. 83*(2), 291-298 (2011).

8. Lah, N. A. C. & Johan, M. R. Facile shape control synthesis and optical properties of silver

nanoparticles stabilized by Daxad 19 surfactant. *Appl. Surf. Sci.* **257**, 7494-7500. (2011).

9. Li, S., Liu, P., & Wang, Q. Study on the effect of surface modifier on self-aggregation behavior of Ag nano-particle. *Appl. Surf. Sci.* **263**, 613-618 (2012).

10. de Faria, A. F., Martinez, D. S. T., Meira, S. M. M., de Moraes, A. C. M., Brandelli, A., Souza Filho, A. G., & Alves, O. L. Anti-adhesion and antibacterial activity of silver nanoparticles supported on graphene oxide sheets. *Colloid. Surface B.* **113**, 115-124. (2014).

11. Sotiriou, G. A., & Pratsinis, S. E. Antibacterial activity of nanosilver ions and particles. *Environ. Sci. Technol.* **44**, 5649-5654 (2010).

12. Khurana, C., Vala, A. K., Andhariya, N., Pandey, O. P., & Chudasama, B. Antibacterial activity of silver: the role of hydrodynamic particle size at nanoscale. *J. Biomed. Mater. Res. A*, **102**, 3361-3368 (2014).

13. Le, A. T., Tam, P. D., Huy, P. T., Huy, T. Q., Van Hieu, N., Kudrinskiy, A. A., & Krutyakov, Y. A. Synthesis of oleic acid-stabilized silver nanoparticles and analysis of their antibacterial activity. *Mat. Sci. Eng. C-Mater.* **30**, 910-916 (2010).

14. Liu, Z., Wang, Y., Zu, Y., Fu, Y., Li, N., Guo, N. & Zhang, Y. Synthesis of polyethylenimine (PEI) functionalized silver nanoparticles by a hydrothermal method and their antibacterial activity study. *Mat. Sci. Eng. C-Mater.* **42**, 31-37. (2014).
